# Revealing the mechanisms behind novel auditory stimuli discrimination: An evaluation of silent functional MRI using looping star

**DOI:** 10.1002/hbm.25407

**Published:** 2021-03-17

**Authors:** Nikou L. Damestani, Owen O'Daly, Ana Beatriz Solana, Florian Wiesinger, David J. Lythgoe, Simon Hill, Alfonso de Lara Rubio, Elena Makovac, Steven C. R. Williams, Fernando Zelaya

**Affiliations:** ^1^ Department of Neuroimaging King's College London London UK; ^2^ ASL Europe, GE Healthcare Munich Germany

**Keywords:** auditory oddball, Looping Star, novel sounds, silent functional MRI, tone discrimination

## Abstract

Looping Star is a near‐silent, multi‐echo, 3D functional magnetic resonance imaging (fMRI) technique. It reduces acoustic noise by at least 25dBA, with respect to gradient‐recalled echo echo‐planar imaging (GRE‐EPI)‐based fMRI. Looping Star has successfully demonstrated sensitivity to the cerebral blood‐oxygen‐level‐dependent (BOLD) response during block design paradigms but has not been applied to event‐related auditory perception tasks. Demonstrating Looping Star's sensitivity to such tasks could (a) provide new insights into auditory processing studies, (b) minimise the need for invasive ear protection, and (c) facilitate the translation of numerous fMRI studies to investigations in sound‐averse patients. We aimed to demonstrate, for the first time, that multi‐echo Looping Star has sufficient sensitivity to the BOLD response, compared to that of GRE‐EPI, during a well‐established event‐related auditory discrimination paradigm: the “oddball” task. We also present the first quantitative evaluation of Looping Star's test–retest reliability using the intra‐class correlation coefficient. Twelve participants were scanned using single‐echo GRE‐EPI and multi‐echo Looping Star fMRI in two sessions. Random‐effects analyses were performed, evaluating the overall response to tones and differential tone recognition, and intermodality analyses were computed. We found that multi‐echo Looping Star exhibited consistent sensitivity to auditory stimulation relative to GRE‐EPI. However, Looping Star demonstrated lower test–retest reliability in comparison with GRE‐EPI. This could reflect differences in functional sensitivity between the techniques, though further study is necessary with additional cognitive paradigms as varying cognitive strategies between sessions may arise from elimination of acoustic scanner noise.

## Graphical abstract

Looping Star is a new multi‐echo functional magnetic resonance imaging technique (fMRI) that reduces acoustic noise of scanning by at least 25dBA, with respect to gradient‐recalled echo echo‐planar imaging (GRE‐EPI)‐based fMRI. We demonstrate, for the first time, that multi‐echo Looping Star is sensitive to the BOLD response during the auditory “oddball” task, an event‐related discrimination paradigm. This is also the first study to evaluate the test‐retest reliability of Looping Star in comparison with GRE‐EPI.

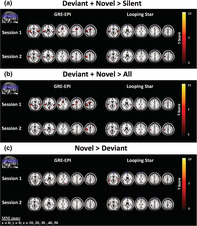

## 1 INTRODUCTION

The inherent acoustic noise of conventional functional magnetic resonance imaging (fMRI), carried out using gradient‐recalled echo echo‐planar imaging (GRE‐EPI), is often intolerably high, commonly achieving sound levels greater than 100dBA (Price, De Wilde, Papadaki, Curran, & Kitney, 2001; Ravicz, Melcher, Kiang, & N., 2000). At these levels, severe hearing damage can occur without ear protection. During GRE‐EPI, this acoustic noise originates primarily from the rapid switching of the frequency encoding magnetic field gradient (from near maximum negative to near maximum positive and vice‐versa), necessary for fast two‐dimensional slice‐by‐slice imaging. This switching induces high‐frequency mechanical vibrations in the scanner hardware, which fall within the acoustic spectrum (Price et al., 2001).

This high acoustic scanner noise impacts the interpretation of the mechanisms behind auditory processing in fMRI studies. For example, Yakunina et al. (2015) showed that the auditory connectivity network differed during a music listening task when a quieter, sparse‐sampling fMRI acquisition technique was used, in comparison with using conventional, noisy, continuous acquisition. Langers, Van Dijk, and Backes (2005) also showed the influence of background scanner noise on the haemodynamic response during auditory tone presentation, using a variable‐length silent gap fMRI acquisition method. Moreover, Gaab, Gabrieli, and Glover (2007) demonstrated the masking effect of scanner background noise on blood‐oxygen‐level‐dependent (BOLD) signal in response to word stimuli. The impact of acoustic scanner noise on auditory processes has been supported by further studies (Healy, Moser, Morrow‐Odom, Hall, & Fridriksson, 2007; Scarff, Dort, Eggermont, & Goodyear, 2004; Shah, Jäncke, Grosse‐Ruyken, & Müller‐Gärtner, 1999).

Background scanner noise can also impose limitations to the generalisability of studies across numerous cohorts. For example, conditions such as tinnitus can include symptoms of hypersensitivity to sound (or hyperacusis) (Baguley, 2003; Chen et al., 2015). fMRI studies have been performed with this cohort, however participants with hyperacusis are often excluded (Araneda et al., 2018; Golm, Schmidt‐Samoa, Dechent, & Kröner‐Herwig, 2013; Han et al., 2018; Hofmeier et al., 2018; Leaver et al., 2011) and in some instances there is difficulty in disentangling whether activity patterns result from the stimulus or the acoustic scanner noise (Ghazaleh et al., 2017; Gu, Halpin, Nam, Levine, & Melcher, 2010; Husain & Schmidt, 2014; Lanting, De Kleine, & Van Dijk, 2009; Leaver, Seydell‐Greenwald, & Rauschecker, 2016; Seydell‐Greenwald et al., 2012). Given that hyperacusis is also heterogeneously prevalent in further cohorts, such as in individuals with autism spectrum disorder (Stiegler & Davis, 2010) and in children (Rosing, Schmidt, Wedderkopp, & Baguley, 2016), mitigating background scanner acoustic noise could greatly improve standardisation across numerous clinical groups.

To date, conventional methods for addressing this high acoustic scanner noise have revolved around the retention of GRE‐EPI acquisition sequences, due to their functional sensitivity and spatiotemporal resolution. One example of this was presented by Seifritz et al. (2006), where they tuned the GRE‐EPI pulse sequence to alter the characteristics of the acoustic noise, but the noise amplitude remained comparable to conventional GRE‐EPI. The primary strategy is to ask participants to employ earplugs during scanning, however effective sound attenuation relies on their correct application, hence there remains a risk of hearing damage (Salvi & Sheppard, 2018; Sheppard, Chen, & Salvi, 2018). Alternative strategies for scanner acoustic noise reduction involve adapting the GRE‐EPI pulse sequence, for example via band‐limited gradient pulses (Hennel, Girard, & Loenneker, 1999) and sparse temporal sampling (Hall et al., 1999). A number of early scanner noise reduction techniques were reviewed by Moelker and Pattynama (2003). Hardware improvements have also been explored, such as gradient coil isolation (Edelstein et al., 2002), and there has recently been increased use of active noise‐cancelling headphones (Dewey et al., 2018; Gabrielsen et al., 2018), although these strategies can be financially costly and therefore not widely applicable across studies. Ultimately, there is no specific optimal workflow or acquisition technique applicable across sites and paradigms to reduce the potential confound and limitations of acoustic scanner noise at its source.

To address these issues, we present the application of a recently developed silent pulse sequence known as Looping Star (Wiesinger, Menini, & Solana, 2019). This technique could mitigate the need for earplugs, improve accessibility to the scanning environment and remove the acoustic noise confound. Looping Star (LS) is based on a technique known as Rotating Ultra‐Fast Imaging Sequence (RUFIS) (Madio & Lowe, 1995), which reduces the effect of vibrations induced by gradient switching by making small incremental changes in the direction (but not the amplitude) of the frequency encoding gradients of the readout. Looping Star is a modification of RUFIS in which a temporal‐multiplexed gradient refocusing mechanism is employed (Wiesinger et al., 2019), allowing the transverse component of the magnetisation to evolve by returning periodically to the centre of k‐space. As a result, it remains sensitive to static T_2_* dephasing as in GRE‐EPI and can achieve multi‐echo acquisition without the need for magnetisation preparation pulses (Solana, Menini, Sacolick, Hehn, & Wiesinger, 2016). A detailed description of the Looping Star methodology can be found in Wiesinger et al. (2019).

To date, Looping Star has proven sensitive to the BOLD response evoked by periodic blocks of sensory stimuli (Damestani et al., 2019a; Wiesinger et al., 2019), visual working memory (Dionisio‐Parra, Wiesinger, Sämann, Czisch, & Solana, 2020) and in the “resting” state (Damestani et al., 2019b; Dionisio‐Parra et al., 2020). However, Looping Star has not been evaluated using event‐related fMRI paradigms, including those of an auditory nature, which are able to probe aspects of cognition in a manner not possible using block‐designs. One such paradigm is the active “oddball” task (Squires, Squires, & Hillyard, 1975), an important auditory discrimination paradigm that has been used extensively in several studies using both EEG (Barry, Kirkaikul, & Hodder, 2000; Justen & Herbert, 2018; Wronka, Kaiser, & Coenen, 2008) and fMRI (Brázdil et al., 2005; Mangalathu‐Arumana, Beardsley, & Liebenthal, 2012). It is particularly relevant in the study of cognitive deficits in participants with autism spectrum disorder (ASD), as these individuals have shown reduced performance during similar tasks when compared with healthy controls (Dawson, Finley, Phillips, & Galpert, 1986; Dawson, Finley, Phillips, Galpert, & Lewy, 1988; Oades, Walker, Geffen, & Stern, 1988).

Importantly, a previous study also highlighted that alternative cognitive strategies were employed by children with ASD during an auditory “oddball” tone discrimination fMRI task (Gomot et al., 2006). This study used an adapted slice‐onset version of conventional GRE‐EPI to account for the acoustic noise limitations. The characterisation of responses to this task using Looping Star, in comparison with this adapted GRE‐EPI acquisition, therefore has clear advantages with respect to its translation to studies involving individuals with ASD, as Looping Star would remove the acoustic noise confound. Demonstrating comparable test–retest reliability of Looping Star would further facilitate this translation. Furthermore, the multi‐echo capabilities of Looping Star are worthy of investigation, given the benefits of echo combination to BOLD signal noise reduction (Kundu, Inati, Evans, Luh, & Bandettini, 2012). These have not yet been evaluated for Looping Star using an event‐related paradigm design.

Hence, our specific aims were:

1. To investigate whether multi‐echo Looping Star is sensitive to the BOLD response elicited during the auditory “oddball” paradigm.
2. To quantitively compare the functional sensitivity of Looping Star with that of a compatible adapted slice‐onset single‐echo GRE‐EPI acquisition, with identical acquisition parameters to those used in the original auditory tone discrimination study (Gomot et al., 2006).
3. To explore the test–retest reliability of the Looping Star and GRE‐EPI acquisitions using two sessions.

We performed the following analyses to address the aims: a) group‐level conventional parametric general linear model (GLM) analyses b) intermodality sensitivity comparisons using percentage signal change and parameter estimates and c) test–retest reliability analysis using intra‐class correlation coefficients (ICC) for each modality between sessions.

## 2 METHODS

### 2.1 Participants

Twelve healthy participants (6 female; mean ± standard deviation age = 31.5 ± 8.0 years; range = 25–54 years) were scanned in two sessions. This number of participants was consistent with that of the healthy control group in the aforementioned study using the same paradigm (Gomot et al., 2006). These sessions were separated by at least 1 week and were no more than 2 weeks apart. All participants took part in both scanning sessions and for the full duration of both sessions. Exclusion criteria involved standard MRI contra‐indications and participants were recruited from within the university (King's College London). Ethical approval was provided under London—Camberwell St Giles REC reference 04/Q0706/72, and informed written consent was obtained from all participants.

### 2.2 Oddball paradigm

For consistency with Gomot et al. (2006), we decided to employ a paradigm with a design identical to that used in their study. The stimuli were presented through the pneumatic MR‐compatible headphones (MR Confon, Cambridge Research Systems). The paradigm involved three tone types (*p* = probability of occurrence), Deviant (*p* = .09), Novel (*p* = .07) and Standard (*p* = .84), played with event duration 80 ms and interstimulus interval 625 ms. Deviant tones were simply frequency‐shifted Standard tones, whereas Novel tones were completely Novel (in terms of pitch and frequency) relative to Standard and Deviant tones (Müller, Jüptner, Jentzen, & Müller, 2002). The beginning of the paradigm was silent for a duration of 10 volumes, then five Standard tones were played. After this, Novel and Deviant tones were played in random order with a minimum of three Standard tones between onsets.

Participants indicated with a button‐box, in the right hand, when either a Deviant or Novel tone was detected, using the same button for both tones. Six silent rest blocks of 10‐second duration were evenly distributed throughout the paradigm. A video of neutral visual distractors, involving animals in natural habitats, was played throughout the paradigm as performed in the original study (Gomot et al., 2006). Deviant and Standard tones were swapped halfway through, as indicated by a screen displaying the command “Swap”, whereby Standard tones became Deviant tones and vice versa, to prevent tone habituation and boredom. Although the Deviant and Standard tones were consistently the same tones when applied, the Novel tones differed for every onset. For further information on the paradigm and characteristics of the tones, we point towards the original study by Gomot et al. (2006), who kindly provided help with the implementation of the paradigm.

Participants' comprehension of the paradigm and hearing ability were tested outside of the scanning facility prior to the first session using a shorter version of the paradigm with frequency‐shifted Standard and Deviant tones, to avoid conditioning effects. These tones were frequency‐shifted by three semitones down from the original tone using version 2.2.2. of the Audacity^®^ recording and editing software (Audacity Team, 2020). Within the scanning sessions, earplugs were provided beneath the pneumatic MR‐compatible headphones. This was to prevent hearing damage during the loud GRE‐EPI acquisition.

Participants self‐reported whether they could hear stimuli presented through the pneumatic MR‐compatible headphones based on whether they could clearly hear the voices of the radiographers through the headphones. The paradigm was then also played through these headphones. To avoid conditioning effects within the session, the tone order in the paradigm differed between Looping Star and GRE‐EPI acquisitions . Otherwise, the same paradigms were used for all participants and for both sessions (i.e., a unique paradigm was assigned to each modality, but not to each session nor each participant). Participant responses during the scans were also monitored via a paradigm‐linked computer to ensure they could consistently hear the paradigm.

### 2.3 fMRI acquisition

Participants wore a pulse oximeter on their forefinger and respiratory belt around their waist to probe any possible differences in physiological parameters (heart rate and respiration rate). A 3T General Electric MR750 Discovery scanner (GE Healthcare, Chicago, IL) with a General Electric 12‐channel receive‐only head coil was used. A standard ADNI (Leung et al., 2015) 1.09 mm in‐plane resolution structural IR‐SPGR image was collected with acquisition parameters: TE = 3.016 ms, TR = 7.312 ms; TI = 400 ms, number of slices = 196, slice‐gap = 1.2 mm, flip‐angle = 11°.

For the fMRI modalities, the same acquisition parameters were used between sessions, and sequence order was pseudo‐randomised between participants and sessions. Acquisition parameters for single‐echo GRE‐EPI were as follows: TE = 27.5 ms, TR = 2.5 s; slice thickness = 4 mm, number of slices = 20, slice‐gap = 1 mm, in‐plane resolution = 3.125 mm, flip‐angle = 82°, 240 volumes, duration = 10 min. As in the case of the study by Gomot et al. (2006), the tone duration (80 ms) and the interstimulus interval of 625 ms, ensured that the stimuli were audible in the time gap between slice read‐outs of the multi‐slice GRE‐EPI scans. The field of view for the GRE‐EPI acquisition did not cover the cerebellum.

To ensure k‐space sampling uniformity in Looping Star, a pseudo‐randomly ordered trajectory was applied (Dionisio‐Parra et al., 2020; Wiesinger et al., 2019). The trajectory was calculated for a nominal spatial resolution of 3.2 mm, including an acceleration factor to produce comparable TR with GRE‐EPI (see Supplementary Material A). This acceleration factor introduces blurring, reducing the effective resolution of the images (Maier et al., 2021), however this pattern is sufficient for fMRI as the centre of k‐space is densely sampled (Kasper et al., 2014). This highlights a benefit of radial acquisition, as other artefacts typical of Cartesian under‐sampling are not introduced.

As a result, the multi‐echo Looping Star acquisition parameters were as follows: multi‐echo TEs = 0 ms, 16.1 ms, 32.2 ms, TR = 2.648 s, equivalent spatial resolution = 3.2 mm, flip‐angle = 3°, readout bandwidth = ±46.875 kHz, 24 spokes per loop, 72 spokes per segment, 1,080 spokes per volume, 240 volumes, duration = 10 min 35 s. For reconstruction of the FID image (TE = 0 ms), missing centre of k‐space samples due to the dead‐time of the receiver were reacquired at the end of the scan by repeating the Looing Star k‐space trajectory at reduced readout gradient amplitude, as described by Wu, Dai, and Ackerman (2007), Wiesinger, Sacolick, and Menini (2016) and Wiesinger et al. (2019).

### 2.4 fMRI preprocessing

Image reconstruction for Looping Star was conducted offline using a “nearest‐neighbour gridding” approach (Wiesinger et al., 2019) in MATLAB (Mathworks, 2019), as the fast Fourier transform cannot be applied directly to non‐Cartesian data. This included density compensation to account for oversampling of the centre of k‐space (Hoge, Kwan, & Bruce Pike, 1997). Furthermore, an inherent property of Looping Star is that the signal from the spoke dephasing outwards is contaminated by the signal from the spoke refocusing inward. This is known as echo‐in/echo‐out interference (Wiesinger et al., 2019). Dionisio‐Parra et al. (2020) demonstrated that addressing this interference by applying a Fermi filter reduced the image resolution, and RF phase‐cycling doubled acquisition time. Instead, optimal combination of the echoes was used to improve the temporal signal‐to‐noise ratio (tSNR) (Kundu et al., 2012).

After reconstruction, the first 10 volumes were removed for both modalities to avoid the influence of effects due to non‐steady state magnetisation. Looping Star images were rescaled by a factor of 10^5^ post‐reconstruction to avoid intensity capping. Looping Star images were cropped using the FSL (Jenkinson, Beckmann, Behrens, Woolrich, & Smith, 2012) command “fslroi” and re‐oriented using SPM‐12 (fil.ion.ucl.ac.uk/spm/). The origins were centred for the FID and echo images to lie on the anterior commissure. Looping Star and GRE‐EPI preprocessing pipelines were almost identical: for single‐echoes they followed the same pipeline, with Looping Star excluding slice‐timing correction since it is a three‐dimensional acquisition technique. For Looping Star, optimal echo‐combination was included in the pipeline but TE‐dependent denoising (DuPre et al., 2019; Kundu et al., 2012) was not applied to Looping Star to ensure consistency of the preprocessing pipelines between modalities. The pipelines are visualised in Supplementary Material B, with further detail provided below.

Looping Star images were bias‐field corrected with ANTS N4‐ITK (Tustison et al., 2010). The high tSNR FID image was used to estimate the motion correction parameters, which were then applied to the multi‐echo image time series. High frequency artefacts were removed from the time series, to avoid errors in echo combination, and concatenation of the FID and echoes was performed in the *z*‐direction using AFNI (Cox, 1996). Optimal echo combination was applied using the “opt_com” command from “tedana.py” in the MEICA (Kundu et al., 2012) pipeline. The pipeline then continued with co‐registration of the FID to the subject's own high‐resolution T1‐weighted scan, which was then applied to the optimally combined dataset, spatial normalisation using unified segmentation (as implemented in SPM‐12) with images saved at 4 mm isotropic resolution and smoothing with an 8 mm FWHM kernel. This smoothing kernel was used to ensure adequate signal‐to‐noise ratio for the Looping Star dataset and as a compromise between the minimum identifiable cluster size and satisfying the Gaussian random field approximation.

Following the same bias‐field correction, the same standard SPM‐12 preprocessing pipeline was applied, though adjusted for single‐echo GRE‐EPI. This included slice‐timing correction, co‐registration to the subject's high‐resolution T1‐weighted scan, spatial normalisation using unified segmentation (as implemented in SPM‐12) with images normalised at 4 mm isotropic resolution and smoothing with an 8 mm FWHM kernel.

### 2.5 fMRI analysis—group level SPM

Single‐subject fixed‐effects and group‐level random‐effects analyses were conducted in SPM‐12, with cluster‐level inference using a primary uncorrected cluster‐forming threshold of *p* < .001 (Woo, Krishnan, & Wager, 2014; Worsley et al., 1996) . Only clusters surviving family‐wise error correction at the cluster‐level (i.e., *p*[FWE_c_] < .05) were deemed significant. The baseline condition (Standard tones) was not modelled explicitly to avoid over‐parameterisation of the general linear model (GLM) and thereby served as an implicit baseline. The first level GLM included modelling the motion parameters as nuisance regressors and modelling three conditions: Deviant, Novel and Silent periods, constructed by convolving regressors encoding the relevant trials with the standard canonical double‐gamma hemodynamic response function. An autoregressive AR(1) model was also used for ReML parameter estimation, used as standard in SPM‐12.

The contrasts interrogated in the first level model were:

- Activity greater during both Novel and Deviant tones over Silent periods (Dev + Nov > Silent)
- Activity greater during both Novel and Deviant tones over Standard tones and Rest (Dev + Nov > All)
- Activity greater for Novel tones than Deviant tones (Nov > Dev) and vice versa (Dev > Nov)
- Activity greater for Novel tones over Deviant, Standard and Rest (Nov > All)
- Activity greater for Deviant tones over Novel, Standard and Rest (Dev > All)

A 128 s high‐pass filter was applied during analysis. MNI co‐ordinates, from the output of SPM‐12, and Brodmann areas were compared using BioImage Suite (Lacadie, Fulbright, Arora, Constable, & Papademetris, 2008).

### 2.6 fMRI analysis—between‐modality comparison

To quantitatively compare, in a general fashion, the functional sensitivity between techniques, a paired *t* test was computed in SPM‐12 between the first level contrast maps of Dev + Nov > Silent (contrast i., Section 2.6) in each session.

To further explore the intermodality difference in functional sensitivity in auditory regions, given the nature of the task and the difference in acoustic load between acquisitions, a Neurosynth (Yarkoni, Poldrack, Nichols, Van Essen, & Wager, 2011)‐derived auditory region of interest (ROI), using the term “auditory” thresholded at *z* = 5, was used as a mask. This mask was applied to the first level contrast maps of Dev + Nov > Silent (i, Section 2.6). The mean parameter estimates, or betas, of the Novel and Deviant tones modelled, as well as the *T*‐scores of the activity maps (see Supplementary Material C) were calculated in this region. The mean T‐score and mean Novel and Deviant parameter estimates were computed across participants. The percentage signal change was also computed within the same ROI, though thresholded at z = 8, using the MarsBaR toolbox of SPM‐12; and its accompanying guidance for batch calculation of the percentage signal change (Brett, Anton, & Valabregue, 2002). The event duration used was zero, and the computed scaling factor within the MarsBaR batch was dependent on the time‐bin used for each modality as detailed in the aforementioned batch.

Normality was tested on the *T*‐scores, beta parameters and percentage signal change values across participants via a Shapiro–Wilk test in version 27.0 of IBM SPSS Statistics (IBM Corp, 2020). The percentage signal change results were therefore quantitatively compared using Spearman's correlation in SPSS to evaluate the consistency of the participant responses between modalities. A Wilcoxon Signed Rank test was also computed in SPSS between modalities for the percentage signal change of each tone between modalities. For the beta parameters of each tone in the auditory ROI and the mean *T*‐score in the auditory ROI, either a Wilcoxon Signed Rank or Paired *T*‐test was computed based on the output of the normality test. All *T*‐tests included a hypothesised mean/median difference of zero and *α* = .05. Statistical significance was determined by a two‐tailed test at a *p*‐value threshold <.05.

### 2.7 fMRI analysis—between‐session differences

#### 2.7.1 Group level intra‐class correlation—within modality

As is customary in quantitative assessments of reliability, the voxel‐wise intra‐class correlation (ICC) analysis (Caceres, Hall, Zelaya, Williams, & Mehta, 2009), using ICC index (3,1), was employed to evaluate between session characteristics for the contrast maps of Deviant + Novel > Silent at group level (i.e., across participants, between sessions, within modality). This was performed to establish the reliability of the activity maps between the scanning sessions, as this method is not sensitive to the mean difference between sessions but rather sheds light on the variability between sessions. The ICC (3,1) (Shrout & Fleiss, 1979) has been proposed specifically for this type of comparison. Its magnitude is calculated using the sum of squares between subjects (BMS) and between sessions (EMS), with k as the number of repeated sessions (Caceres et al., 2009), seen in Equation (1).

(1) $$\mathrm{ICC}\left(3,1\right)=\frac{\mathrm{BMS}-\mathrm{EMS}}{\mathrm{BMS}+\left(k-1\right)\mathrm{EMS}}$$

An ICC of 1 indicates exceptionally high reliability between sessions as the between session variability would be close to zero (i.e., the error sum of squares would be negligible). On the other hand, the ICC becomes negative as the size of the between‐sessions variance regression becomes larger than the between‐subject variance. An ICC close to −1 therefore, (the other extreme), indicates exceptionally poor between session reliability; and that this term would be significantly larger than the variability between subjects (i.e., the between‐subject sum of squares is close to zero).

The contrast maps were first masked with a grey matter mask (grey matter tissue prior from SPM‐12, see Supplementary Material C). A task‐related network mask was then defined from the first session for each modality, by means of a low *T*‐score threshold of 1. A low threshold was used to account for the difference in amplitude of the *T*‐scores between techniques, preventing large clusters from being more prevalent in one modality than another. The median ICC score for all voxels within the mask was calculated. When calculating the network mask, grand mean scaling and global calculation were omitted.

#### 2.7.2 Intra‐voxel reliability—within modality

The ICC toolbox can also be applied to test the consistency of the signal distribution within an ROI across sessions. This produces a region ICC for each subject (i.e., across sessions, within modality) and is known as the intra‐voxel reliability (ICC_v_). This differs from a typical voxel‐wise ICC where the reliability of the signal across sessions is determined separately for each voxel.

The intra‐voxel reliability was calculated for each subject within an auditory ROI, generating an intra‐voxel ICC_v_ value for each participant. This auditory ROI was computed across a Neurosynth (Yarkoni et al., 2011)‐derived auditory ROI (see Supplementary Material C), using the term “auditory” and thresholded at *z* = 5. In this case, Equation (1) is applied for each individual subject using the contrast values of the voxels within this auditory ROI as stated by Caceres et al. (2009). As this work suggests, the intra‐voxel reliability then measures the total variance explained by the intra‐voxel variance, testing the consistency of the spatial characteristics of the BOLD signal distribution in this ROI to infer differences between subjects. The ROI was applied to the contrast maps for Deviant + Novel > Silent for each participant and across sessions.

#### 2.7.3 Comparison of intra‐voxel reliability—between modality

To evaluate the differences in ICC_v_ between modalities, the mean and standard deviation of the outputted ICC_v_ from the intra‐voxel reliability calculation was computed across participants. Upon computing a Shapiro–Wilk normality test, a Wilcoxon Signed Rank Test was computed between modalities to compare medians of the ICC_v_ values, given that the same population produced ICC_v_ scores for the two modalities, using SPSS. Statistical significance was determined by a two‐tailed test at a *p*‐value threshold <0.05. Although Bland–Altman plots (Bland & Altman, 1999) have been used in some studies to explore reliability, these were not used in our work as the literature indicates that they are more appropriate when assessing direct replication of quantitative absolute measures, which is not the case of beta parameters in fMRI analysis.

### 2.8 Image quality measures

Temporal signal‐to‐noise ratio (tSNR) was calculated as outlined by Friedman and Glover (2006). The mean signal across time per voxel was computed and divided by its corresponding standard deviation after second‐order polynomial de‐trending (i.e., the standard deviation of the residuals). The images used were those preprocessed including all steps up to spatial normalisation (i.e., excluding smoothing), to produce the average tSNR value across participants. The tSNR was also calculated within a grey matter mask (grey matter tissue prior from SPM‐12, see Supplementary Material Figure C). This measure avoided artefacts in the average tSNR images that result from differences in brain structure. A difference map was produced by dividing the difference between the modality tSNR maps within‐session by the sum of the maps and multiplying this result by 100.

### 2.9 Sound level measurements

A direct sound level measurement was taken by attaching the Casella 62X (Casella Solutions, UK) sound meter on a cylindrical phantom at the axial isocentre of the magnet bore and taking the mean LCpeak and LAeq values across 15 s (approximately 5 volumes) for each scanning sequence.

## 3 RESULTS

### 3.1 Looping Star acoustic noise and image quality characteristics

Table 1 shows the in‐bore sound amplitude measures inside the scanner, indicating that Looping Star was less than 10dBA louder than the ambient scan room noise and 27dBA quieter than GRE‐EPI. This is a substantial difference, particularly since acoustic noise is measured on a logarithmic scale.

**TABLE 1:** Average sound level measures over a duration of 15 s of scanning from bore isocentre of each acquisition modality

Acquisition	LAeq (dBA)	LCpeak (dBC)
GRE‐EPI	98.0	112.9
Looping Star	71.0	102.8
Ambient scanner room, no scan	64.0	85.7

**FIGURE 1:**
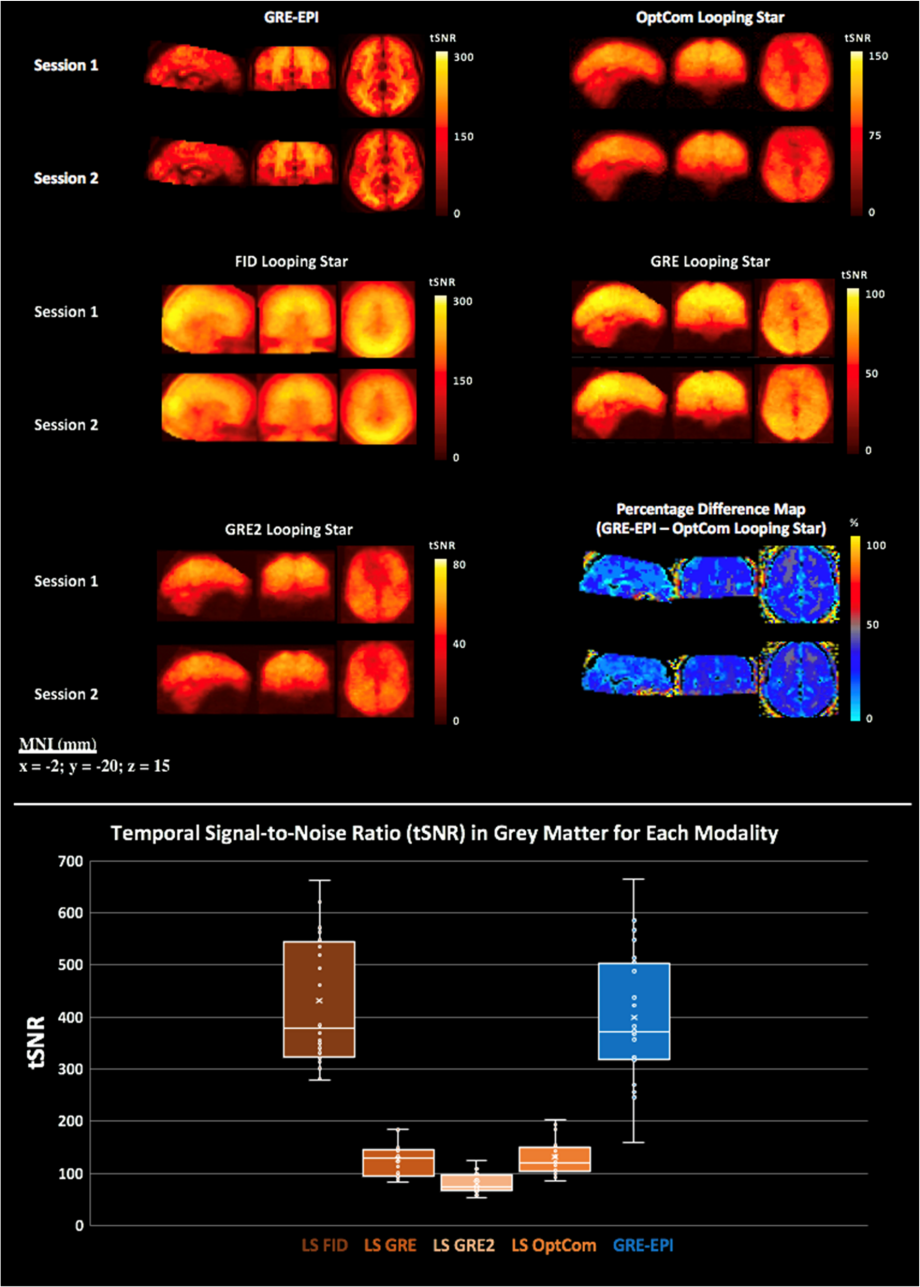
(top) Mean temporal signal‐to‐noise‐ratio (tSNR) maps, calculated across participants for each modality and for separate echoes (free induction decay—FID, Echo 1—GRE, Echo 2—GRE2) and the optimally combined echoes (OptCom) in Looping Star (LS). Datasets were realigned and spatially normalised prior to computation of the tSNR. Percentage difference maps between optimally combined Looping Star and GRE‐EPI for each session are also shown at the bottom right. (bottom) tSNR value distribution across subjects and sessions within grey matter mask for each modality. Slice (mm = millimetres) in MNI space provided

The tSNR results can be seen in Figure 1 for each individual echo and for the optimally combined temporal series from Looping Star, compared with GRE‐EPI. tSNR overall was lower in Looping Star compared with GRE‐EPI, evident both visually in the whole brain (Figure 1, top) and in the quantitative values in grey matter (Figure 1, bottom). The distribution of tSNR values was narrower for the echoes and optimally combined echoes of Looping Star than in GRE‐EPI. The percentage difference map indicated less than 50% difference between optimally combined Looping Star and GRE‐EPI, whereas higher differences could be seen in white matter, which is likely driven by the different tissue relaxation characteristics between techniques. A figure of the raw images has also been provided (Supplementary Material D).

### 3.2 Physiological and behavioural responses

The mean heart rate and respiratory volume per time across the acquisition did not demonstrate any significant differences between modalities (Supplementary Material E). Participants were over 86% accurate on average for both modalities and sessions, indicating satisfactory cognitive engagement with the paradigm. Only one participant had lower than 86% accuracy during GRE‐EPI Session 2, but they were still over 76% accurate. There was no evidence of poorer performance accuracy after the tones were swapped (Supplementary Material E).

### 3.3 Whole‐brain voxel‐wise GLM random‐effects analysis

**FIGURE 2:**
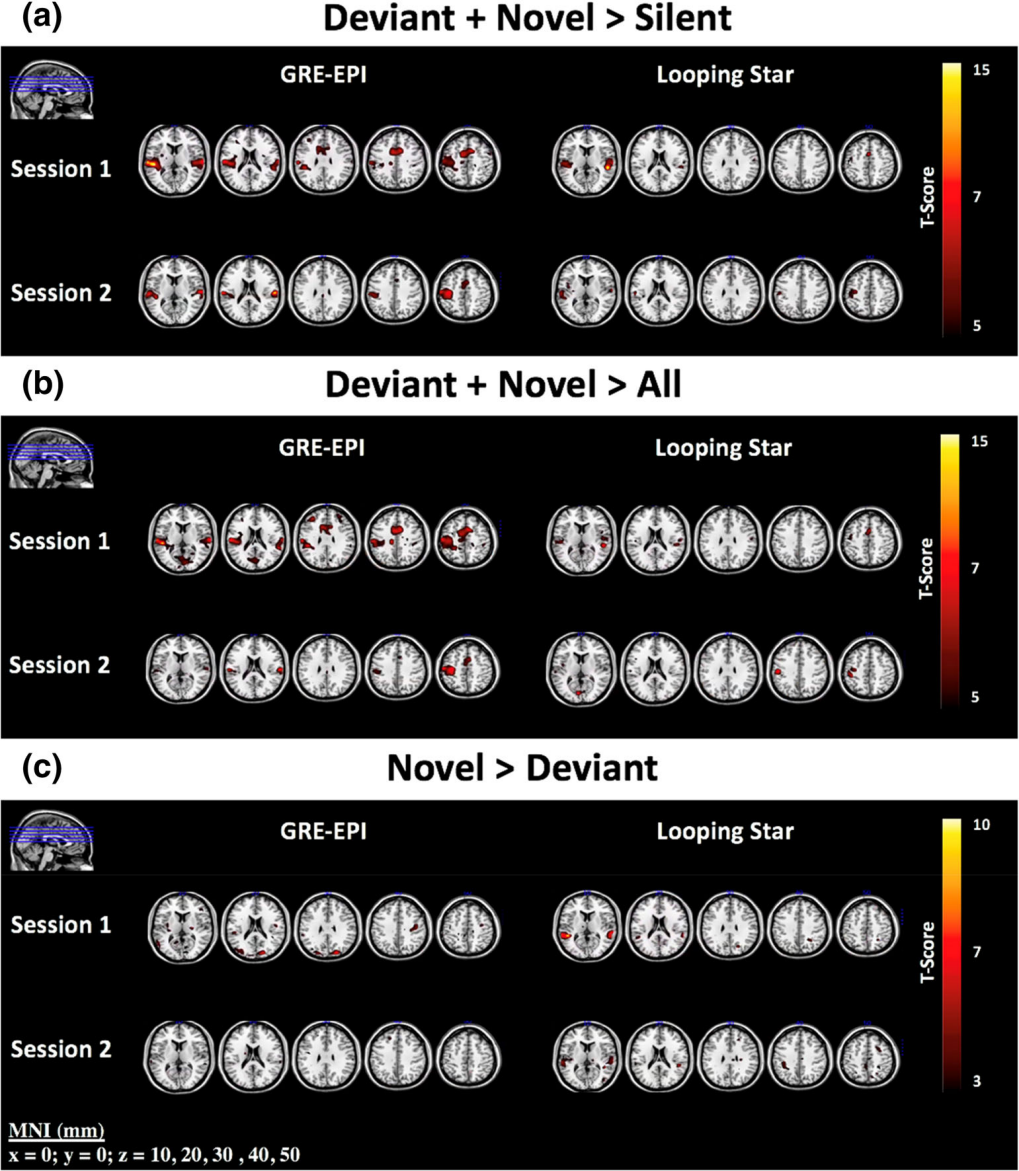
Parametric activity maps for the contrasts (a) Deviant + Novel > Silent, (b) Deviant + Novel > All other onsets and (c) Novel > Deviant. Regions of comparable activity can be seen for (a) and (b), whereas (c) highlights that only Looping Star Session 1 detects an auditory response for the contrast. Slices shown are also visualised in top left corner of images. Overlaid on ch2 image (Holmes et al., 1998) in MRICRON (Rorden & Brett, 2000). Statistics at *p* < .001 uncorrected can be seen in Table 2. Slice (mm = millimetres) in MNI space provided

Since the standard tone events served as an implicit baseline, we evaluated the overall sensitivity to auditory stimuli between modalities, using the contrasts: Deviant + Novel > Silent (rest blocks) and Deviant + Novel > All other blocks. Figure 2a,b shows that Looping Star and GRE‐EPI were both sensitive to the responses to non‐standard tones. Activation was observed in the same Brodmann areas (BA) identified by the original study (Gomot et al., 2006), namely the anterior transverse temporal area (BA 41) and the posterior superior temporal gyrus (BA 22) (Table 2). Significant activity was also identified in both modalities within the motor cortex (BA 6) and somatosensory cortex (BA 1). No significant results were identified for the contrast Deviant > Novel with either technique, however Figure 2c demonstrates the regions more responsive to Novel trials than Deviant trials. Only the Looping Star Session 1 data yielded a statistically significant BOLD response to this contrast in an auditory region.

**TABLE 2:** SPM statistics table of results for parametric analysis at primary uncorrected cluster‐forming threshold ( *p* < .001 unc.) across different contrasts for each session and modality

Contrast name	Modality	MNI co‐ordinates (*x*,*y*,*z* in mm)	Brodmann area	Cluster‐level *p*(FWE)‐value	*T*‐score	Cluster size
Deviant + Novel > Silent	GRE‐EPI Session 1	−46 −28 10	41	<10^−3^ **	15.86	2,325
		66 −20 10	41	<10^−3^ **	9.33	477
		42 −32 46	40	0.027*	4.26	40
		34 28 26	9	0.003*	4.06	67
	GRE‐EPI Session 2	58 −20 18	40	<10^−3^ **	13.52	217
		−62 −20 18	1	<10^−3^ **	13.06	616
		−6 0 58	6	<10^−3^ **	9.56	278
		6 −84 −10	18	0.064	8.30	33
		−14 24 6	N/A	0.004*	7.56	73
	Looping Star Session 1	50 −32 14	41	<10^−3^ **	16.91	242
		2 −76 −22	N/A	<10^−3^ **	11.15	130
		34 −52 −26	N/A	0.197	10.50	12
		−2 4 50	6	<10^−3^ **	9.16	91
		−34 −4 58	6	<10^−3^ **	8.33	127
		−46 6 −6	13	<10^−3^ **	7.77	222
	Looping Star Session 2	−58 −20 22	1	<10^−3^ **	9.60	542
		6 −64 −14	N/A	<10^−3^ **	10.02	329
		62 −12 6	41	0.006*	8.96	36
		50 16 −10	N/A	0.001*	7.92	56
		2 4 66	6	0.010*	5.73	32
Deviant > All	GRE‐EPI Session 1	−50 −28 10	41	<10^−3^ **	14.43	3,166
		34 8 34	13	<10^−3^ **	6.41	165
		54 8 −2	44	0.005*	6.05	45
	GRE‐EPI Session 2	62 −16 18	40	0.001*	14.07	71
		10 −92 −6	17	<10^−3^ **	12.93	77
		−58 −16 50	N/A	<10^−3^ **	11.63	328
		2 12 58	6	<10^−3^ **	10.05	236
		−62 −20 22	1	0.001*	8.86	74
		10 28 −2	N/A	<10^−3^ **	7.92	123
	Looping Star Session 1	−2 0 66	6	<10^−3^ **	9.91	226
		−50 −24 10	40	<10^−3^ **	9.93	50
		−54 4 −2	6	0.014*	8.48	26
		54 −20 22	N/A	<10^−3^ **	8.07	85
		30 0 −6	49	<10^−3^ **	7.52	120
		2 −76 −22	N/A	<10^−3^ **	7.48	134
	Looping Star Session 2	−50 −20 42	1	<10^−3^ **	9.20	130
		−6 −40 −22	N/A	0.121	7.90	15
		−6 −80 10	17	<10^−3^ **	7.60	95
Novel > All	GRE‐EPI Session 1	−58 −20 18	1	<10^−3^ **	13.96	2,775
		−10 −48 −2	N/A	<10^−3^ **	10.24	890
		−42 −36 −10	N/A	0.056	9.66	30
		−34 36 26	N/A	0.001*	8.86	88
		30 32 26	9	<10^−3^ **	7.85	101
	GRE‐EPI Session 2	−46 −20 58	1	<10^−3^ **	10.86	333
		−58 −24 14	40	0.004*	10.15	89
		−2 0 62	N/A	<10^−3^ **	8.87	212
		62 −20 18	40	0.004*	8.51	90
	Looping Star Session 1	54 −32 14	41	<10^−3^ **	11.55	255
		−2 −72 −18	N/A	<10^−3^ **	7.91	137
		−2 0 66	6	<10^−3^ **	7.43	72
		−43 4 −6	13	0.001*	6.88	52
		−62 −20 10	1	<10^−3^ **	6.70	98
		−14 −20 2	50	0.018*	6.55	26
		−18 −28 78	N/A	<10^−3^ **	6.31	186
	Looping Star Session 2	−54 −24 42	40	<10^−3^ **	12.15	455
		58 −16 6	41	0.001*	9.77	49
		14 −44 −14	N/A	<10^−3^ **	8.22	265
		54 12 −6	N/A	0.005*	7.44	37
		54 −24 30	40	0.044*	7.27	22
		−46 12 18	44	0.001*	7.11	49
Novel > Deviant	GRE‐EPI Session 1	22 −84 26	19	0.009*	7.11	35
		−18 −92 26	18	0.022*	5.73	28
	GRE‐EPI Session 2	−18 −8 22	48	0.907	5.70	2
	Looping Star Session 1	−46 −40 10	22	0.004*	10.29	37
		58 −32 15	22	0.001*	6.91	47
	Looping Star Session 2	42 −44 14	N/A	0.218	6.47	12
Deviant > Novel	GRE‐EPI Session 1	14 −64 46	N/A	0.946	4.87	2
	GRE‐EPI Session 2	NSC	NSC	NSC	NSC	NSC
	Looping Star Session 1	22 12 14	N/A	0.981	4.19	1
	Looping Star Session 2	−38 −36 −6	N/A	0.974	4.36	1
Deviant + Novel > All	GRE‐EPI Session 1	−50 −28 10	41	<10^−3^ **	13.83	4,099
		54 12 −2	44	0.039*	6.50	32
		30 −4 18	N/A	0.036*	5.73	33
	GRE‐EPI Session 2	2 12 58	6	<10^−3^ **	11.88	261
		62 −20 18	40	<10^−3^ **	11.40	110
		−46 −20 58	1	<10^−3^ **	11.05	370
		2 −84 −10	18	0.11	10.40	57
		−6 28 2	N/A	0.001*	9.24	90
		−62 −20 18	1	0.001*	8.92	97
	Looping Star Session 1	−2 0 66	6	<10^−3^ **	12.54	313
		50 −32 14	41	<10^−3^ **	11.05	307
		2–76 −22	N/A	<10^−3^ **	10.63	180
		−62 ‐20 6	41	<10^−3^ **	8.04	106
		−54 8–6	22	<10^−3^ **	7.43	50
	Looping Star Session 2	−50 −20 42	1	<10^−3^ **	13.76	275
		−6 −80 10	17	<10^−3^ **	9.84	252
		50 16–10	N/A	0.002*	7.28	44
		−46 12 18	44	0.003*	6.96	41
		−6 −40 −22	N/A	0.035*	6.55	23

Abbreviations: N/A, outside of defined Brodmann Area; NSC, no significant clusters.

* Cluster‐level *p*(FWE) < .05.

** Cluster‐level *p*(FWE) < .001.

### 3.4 Quantitative comparison between modalities (within session)

**FIGURE 3:**
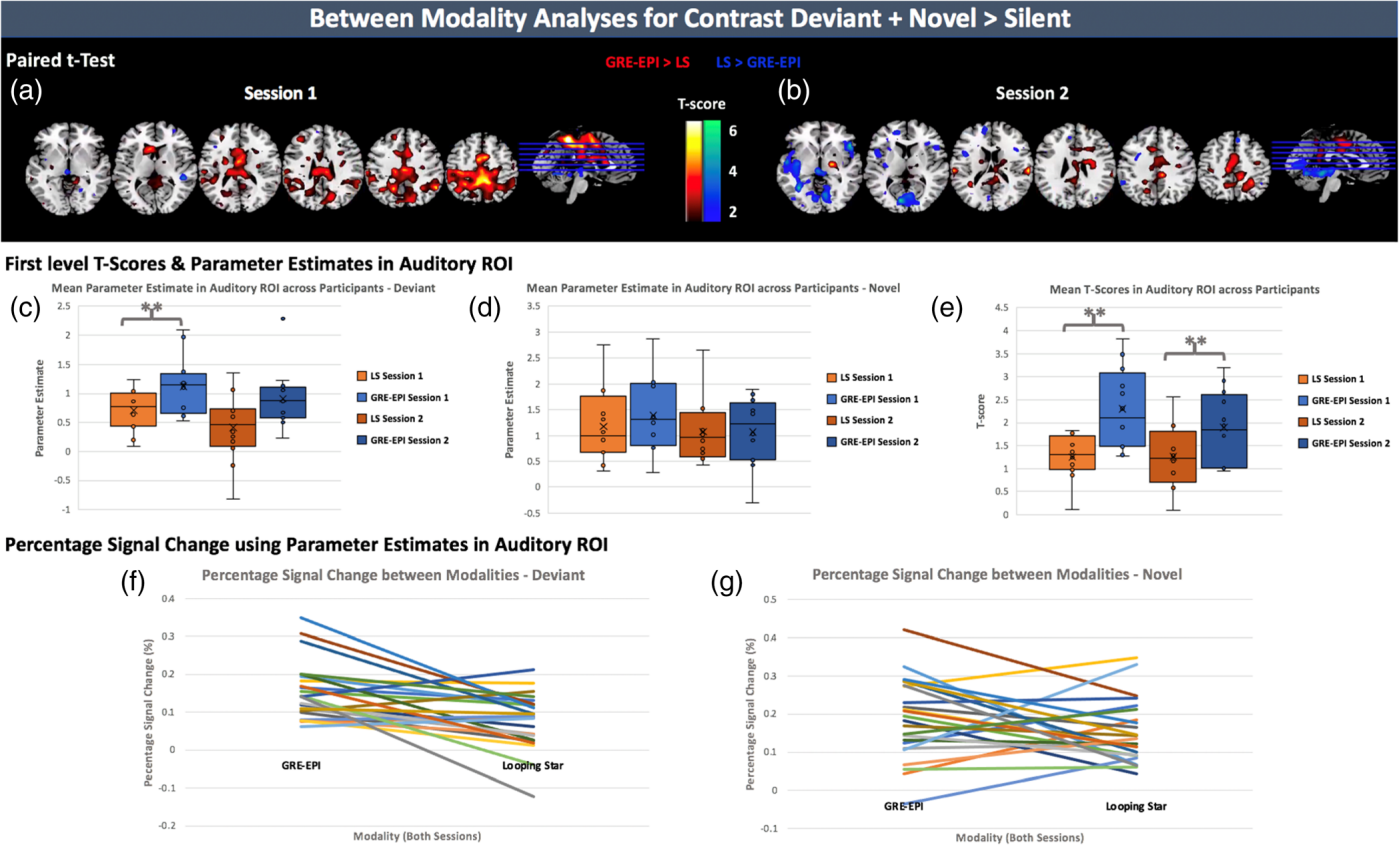
Between modality analyses using Deviant + Novel > Silent contrast maps. (top) Bidirectional results of paired *T*‐test between first level contrast maps of all participants. (a) Session 1 comparison and (b) Session 2 comparison. Overlaid on ch2 image (Holmes et al., 1998) in MRICRON (Rorden & Brett, 2000). (middle) An auditory ROI was used to mask parameter estimate (beta) maps and the mean parameter estimate was calculated for the regressors of the (c) Deviant onsets and (d) Novel onsets and plotted for all participants. (e) The mean *T*‐score was calculated from the first level T‐maps for the contrast, and plotted for each participant after auditory ROI masking. (bottom) Percentage signal change based on parameter estimates in auditory ROI, withall sessions included and plotted for each modality. Pattern of difference between modality shown for (f) Deviant and (g) Novel tones. LS, Looping Star. **p(two‐tailed) < .05. Accompanying statistics seen in Tables 3, 4, 5

Figure 3 demonstrates the results of the quantitative comparisons between modalities, within each session. The intermodality paired *t* tests of the statistical maps for each session (Figure 3a,b) highlight regions of differences in activity. Table 3 provides the accompanying statistics. Within Session 1, only motor cortices (i.e., BA 4 and BA 6) presented statistically significant higher activity for GRE‐EPI relative to Looping Star.

**TABLE 3:** SPM statistics table of results for parametric paired *T*‐test at primary uncorrected cluster‐forming threshold (*p* < .001 unc.) for activity maps of contrast Deviant + Novel > Silent across participants for each session

Paired *T*‐test	MNI co‐ordinates (*x*,*y*,*z* in mm)	Brodmann area	Cluster‐level *p*(FWE)‐value	*T*‐score	Cluster size
GRE‐EPI > LS Session 1	−34 −28 62	4	0.001*	8.72	50
	2 −44 58	N/A	<10^−3^ **	8.44	176
	6 4 46	6	0.002*	5.30	45
GRE‐EPI > LS Session 2	62 −24 18	40	0.578	5.81	6
LS > GRE‐EPI Session 1	14 −60 −10	17	0.299	4.69	10
LS > GRE‐EPI Session 2	10 −52 −6	N/A	<10^−3^ **	6.54	79

Abbreviation: LS, Looping Star; N/A, outside of defined Brodmann Area.

* Cluster‐level *p*(FWE) < .05.

** Cluster‐level *p*(FWE) < .001.

The statistical comparisons of the different measures in an auditory ROI presented in Figure 3c–e are shown in Table 4. We found a statistically significant difference in the mean T‐score within the auditory ROI between GRE‐EPI and Looping Star for both sessions. We also found a statistically significant difference within the auditory ROI for the mean parameter estimates of the Deviant tones in Session 1. There was no significant difference for the parameter estimates of the Novel tones. This was also consistent with the percentage signal change results (Figure 3f,g). Table 5 shows the results of the intermodality Spearman's correlation, which was between 0.08 < *r* < 0.32, as well as the *T*‐tests between the percentage signal change values. Only Deviant tones in Session 1 provided a statistically significant intermodality difference in percentage signal change.

**TABLE 4:** Wilcoxon Signed Rank Test or paired *T*‐test results across different intermodality measures within the auditory ROI

Test variables	Wilcoxon signed rank/paired *T*‐test two‐tailed *p*‐value	Wilcoxon signed rank/paired *T*‐test *T*‐score
Mean *T*‐score in auditory ROI^†^ GRE‐EPI—LS Session 1	0.004*	3.648
Mean *T*‐score in auditory ROI^†^ GRE‐EPI—LS Session 2	0.043*	2.284
Mean Deviant beta parameter in auditory ROI^†^ GRE‐EPI—LS Session 1	0.022*	2.677
Mean Deviant parameter in auditory ROI GRE‐EPI—LS Session 2	0.060	−1.883
Mean Novel beta parameter in auditory ROI^†^ GRE‐EPI—LS Session 1	0.442	0.798
Mean Novel beta parameter in auditory ROI GRE‐EPI—LS Session 2	0.814	−0.235

* *p* < .05.

^†^ Parametric paired *T*‐test.

**TABLE 5:** Intermodality Spearman's correlation and Wilcoxon Signed Rank Test results between percentage signal change values within the auditory ROI

Correlation pair	Spearman's *r*	Wilcoxon signed rank *T*‐score	Wilcoxon signed rank two‐tailed *p*‐value
GRE‐EPI—Looping Star Session 1, Deviant tones	0.252	−2.510	0.012*
GRE‐EPI—Looping Star Session 2, Deviant tones	0.133	−2.197	0.028
GRE‐EPI—Looping Star Session 1, Novel tones	−0.056	−1.334	0.182
GRE‐EPI—Looping Star Session 2, Novel tones	0.315	−0.471	0.638
GRE‐EPI—Looping Star All sessions, Deviant tones	0.320	−3.343	<0.001**
GRE‐EPI—Looping Star All sessions, Novel tones	0.088	−1.257	0.209

* *p* < .05.

** *p* < .001.

### 3.5 Quantitative comparison between sessions (within modality)

**FIGURE 4:**
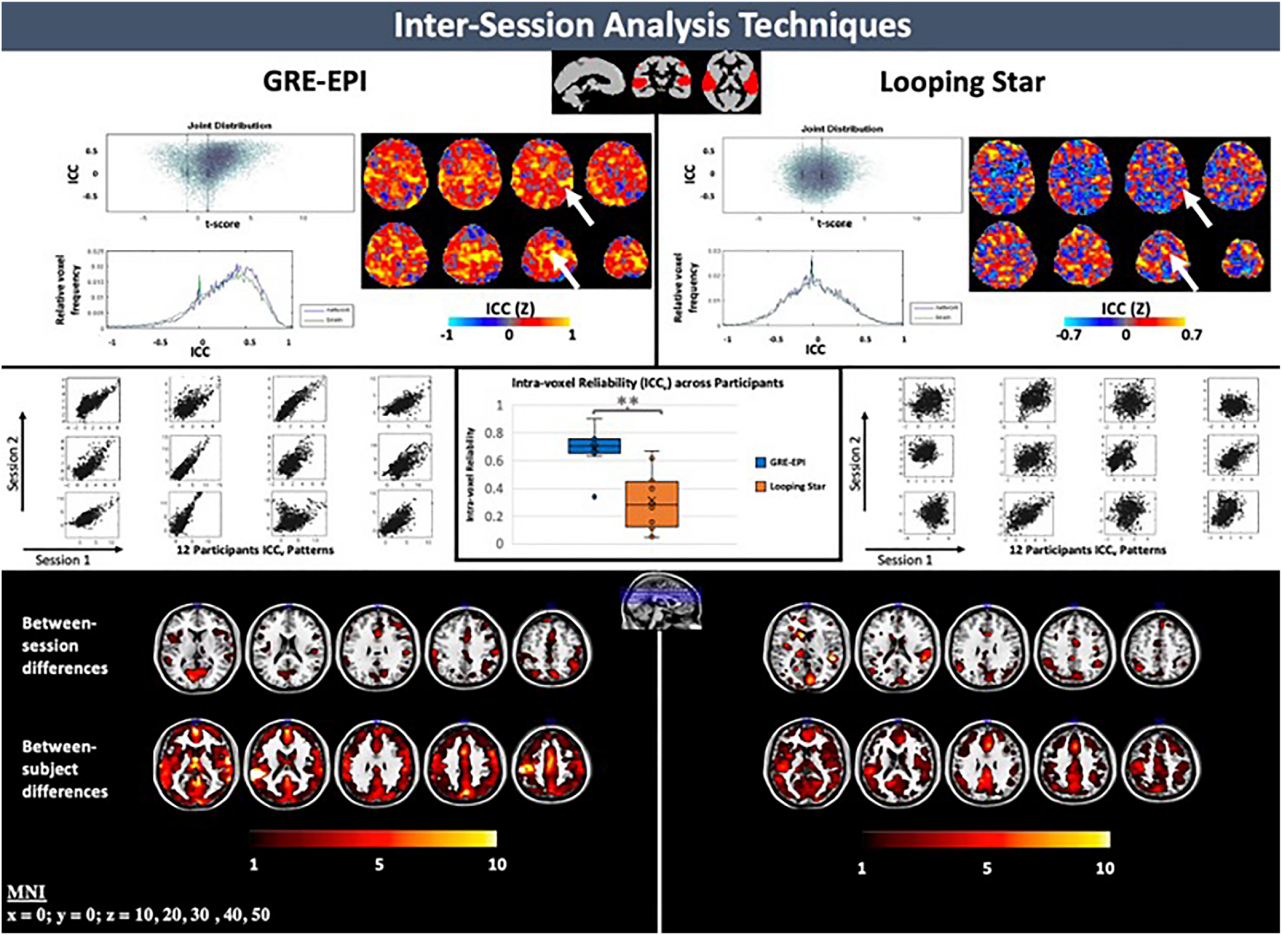
(top) Plots of intra‐class correlation coefficient (ICC) versus *T*‐score, relative voxel frequency versus ICC and ICC *z*‐score map for each modality. Arrows indicate regions with consistently high z‐scores between modalities. (centre) Intra‐voxel reliability (ICC_v_) plots for each participant in an auditory region‐of‐interest (ROI) (top, centre) can be seen with accompanying box‐and‐whisker plot of the outputted ICC_v_ valuesacross participants. Significant differences between intermodality intra‐voxel reliability was identified, where *p*(two‐tailed) = .002 (**p < .05). (bottom) Between‐session and between‐subject difference maps outputted from ICC analysis. Overlaid on ch2 image (Holmes et al., 1998) in MRICRON (Rorden & Brett, 2000). Slice (mm = millimetres) in MNI space provided. Scale of ICC *z*‐score maps adjusted to account for functional sensitivity differences between modalities

Intersession differences, evident in the random‐effects analyses, were more specifically characterised with an ICC analysis. Figure 4 (top) summarises the group level ICC results within modality, where the ICC values are consistently high in task‐related regions, such as the auditory and motor cortices, in both modalities for the contrast maps of Deviant + Novel > Silent. However, the spatial extent with high ICC, in the regions detected with Looping Star, was much smaller than in GRE‐EPI and negative ICC values were seen in regions from the Looping Star data, outside of task‐related regions. This was not the case in GRE‐EPI data. A more skewed joint distribution (towards higher ICC values) was seen in GRE‐EPI between activation *T*‐score and ICC score in comparison with Looping Star.

An independently derived functional ROI of the auditory cortex (Supplementary Material C), was used to calculate the intra‐voxel reliability (ICC_v_) between sessions for each subject, specifically testing between session consistency in an auditory region in each individual. Significantly higher ICC_v_ values were identified using this method on average across subjects in this auditory ROI for GRE‐EPI than Looping Star (Figure 4, centre), where *p*(two‐tail) = .002. There was no evidence of one participant producing particularly low ICC_v_ values in both modalities (i.e., having low ICC_v_ in both modalities).

To further evaluate the ICC results, the between‐subject sum of squares and between‐session sum of squares outputs from the ICC analysis, which are calculated from the activity maps, were explored (Figure 4, bottom). Both modalities demonstrated clusters of low spatial extent with high between‐session variance in the visual and auditory cortices. Although both modalities demonstrated high between‐subject variance within the frontal lobe and auditory cortex, GRE‐EPI demonstrated higher between‐subject variance than Looping Star in the auditory cortex and along the longitudinal fissure.

## 4 DISCUSSION

### 4.1 Summary of results

With respect to our key aims, we demonstrated that (a) multi‐echo Looping Star is sensitive to the BOLD response elicited during the auditory oddball paradigm, (b) multi‐echo Looping Star has comparable sensitivity for novel tone discrimination in auditory regions, relative to a GRE‐EPI acquisition with identical parameters to Gomot et al. (2006), though intermodality differences were also identified, and (c) multi‐echo Looping Star has lower test–retest reliability than GRE‐EPI. We also observed some limitations in the Looping Star technique, such as its reduced tSNR, driven in part by the need to employ a very low excitation flip angle in this modality.

### 4.2 Physiological and behavioural data

On average, there were no differences in the mean heart rate and respiration volume per sampling time across participants for the duration of the different scans. This suggests that overall there were no additional systemic effects imposed by either imaging modality across the paradigm duration. Behaviourally, participants performed very well with regards to detecting the Deviant and Novel tones during both modalities. This is unsurprising as the GRE‐EPI acquisition parameters used were identical to that used in the original study by Gomot et al. (2006), especially ensuring that the EPI slice acquisition train contained the appropriate delays for the tones to be heard by the participants. Our behavioural data also indicated that subjects remained attentive and alert throughout the scans.

### 4.3 Whole‐brain voxel‐wise random‐effects analysis

In general, our results demonstrated good agreement with those of Gomot et al. (2006). The main effect of Deviant and Novel tones versus Silence (Deviant + Novel > Silent) demonstrated consistent bilateral activity in both GRE‐EPI and Looping Star sessions, providing evidence that Looping Star is sensitive to the BOLD response in event‐related auditory paradigms. Right hemisphere involvement of BA 41 was seen in both modalities, which may be linked to the right hemisphere involvement in attention‐related processes (Müller et al., 2002; Stevens, Calhoun, & Kiehl, 2005). Similar activity patterns were also seen in the response to Deviant + Novel > All, which addressed potential issues with the Silent condition likely being an unstable baseline due to its short duration.

The separate contrasts of Deviant > All and Novel > All also demonstrated significant results in auditory regions for both modalities, but there were no significant results for the contrast Deviant > Novel. This could indicate that higher attention was paid to the Novel tones, eliciting a higher amplitude response in spatially overlapping regions relative to Deviant tones. This is supported by the functional overlap for Deviant and Novel tones seen here and by Gomot et al. (2006), alongside the proximity of the overlap of auditory loci and the prevalence of attention‐driven modulations observed in a meta‐analysis by Alho, Rinne, Herron, and Woods (2014). A key intermodality difference that should be explored in depth with a larger cohort was that only Looping Star Session 1 revealed significantly greater activity in response to Novel trials compared to Deviant stimuli (Novel > Deviant) in an auditory region. There were no behavioural motivations for this to be the case, therefore future studies may benefit from exploring potential differences in cognitive engagement, perhaps using a different oddball paradigm. This would inform whether the differences we observed are linked to the reduced auditory load in Looping Star.

### 4.4 Measurement of between‐modality differences

The intermodality paired *t* test for the contrast of Deviant and Novel tones versus Silence (Deviant + Novel > Silent) indicated that there were no statistically significant differences in activity in auditory regions, but differences were present in motor cortices. However, significant activity in the motor cortex was indeed identified in both modalities, therefore these differences are likely related to functional sensitivity differences between techniques that lead to more localised responses in Looping Star. Significant differences in percentage signal change were only identified for the parameter estimate of the Deviant stimuli, which were much lower in Looping Star compared to GRE‐EPI. To verify whether this could be linked to the difference in auditory demand, application of Looping Star with a paradigm exploring repetition priming (Bergerbest, Ghahremani, & Gabrieli, 2004) may be of benefit.

### 4.5 Measurement of between‐session reliability

The ICC results overall indicated lower reliability of Looping Star activity maps for the contrast Deviant + Novel > Silent compared with those of GRE‐EPI. This was demonstrated by the following: (a) the joint distribution indicated a strong relationship between T‐score and ICC for GRE‐EPI, that is, a strong relationship between activity and repeatability, that was not apparent for Looping Star, (b) higher ICC values were seen in the auditory and motor cortex for GRE‐EPI and Looping Star relative to the rest of the brain, though Looping Star demonstrated negative ICC values across the cortex, outside of the auditory regions, unlike GRE‐EPI, suggesting high between‐session variance, and (c) higher intra‐voxel reliability (ICC_v_) was seen in the auditory ROI for GRE‐EPI than Looping Star on average across participants, therefore the signal distribution is more consistent in GRE‐EPI than Looping Star within the auditory ROI. The lower tSNR and smaller identified clusters in Looping Star could be a contributing factor to this intersession difference, though cognitive links to acoustic background noise have been identified in previous studies that could also contribute (Cho, Chung, Lim, & Wong, 1998; Kiehl & Liddle, 2003; Novitski et al., 2003; Seifritz et al., 2006; Wolak et al., 2016). Future replicability and repeatability studies should aim to disentangle these effects.

### 4.6 Limitations and future work

It is important to emphasise that our intention was to perform the first evaluation of multi‐echo silent fMRI in an event‐related context, and we acknowledge that a larger cohort would improve the generalisation of these findings. Limitations regarding the paradigm design, such as the duration of the resting blocks being barely longer than the haemodynamic response, were unavoidable as we aimed to replicate the paradigm used by Gomot et al. (2006). We did, however, adapt the original general linear model by deciding against modelling the Standard tones (Gomot et al., 2006) to avoid over‐parameterisation. Our desire to reproduce the conditions of the study of Gomot et al as much as possible, also meant that we did not acquire multi‐echo GRE‐EPI data and so we were limited in our comparisons. There were also some inherent limitations in the pulse sequence design of the version of Looping Star that we employed, which prevented both faster imaging and higher tSNR. These have been outlined in detail by Dionisio‐Parra et al. (2020).

There is scope to further characterise Looping Star for targeting specific optimisation strategies. Such avenues include evaluating the impact of spatial blurring induced during acquisition, assessing whether anatomical configuration interacts with certain acquisition parameters, and exploring the impact of physiology on this three‐dimensional acquisition. Future studies could also capitalise on the reduced impact of inflow effects in Looping Star given the absence of slice selection. Furthermore, alternative reconstruction schemes, beyond the one employed here for Looping Star, may be more appropriate in future studies. Such techniques include compressed sensing and low‐rank reconstruction, which employ under‐sampled k‐space trajectories (Chiew et al., 2015; Holland et al., 2013; Zong, Lee, Poplawsky, Kim, & Ye, 2014).

## 5 CONCLUSIONS

Looping Star provides a useful, near‐silent MRI acquisition alternative that mitigates the limitations produced by the high acoustic noise of GRE‐EPI, providing a “real‐world” scenario for functional neuroimaging. It also removes the reliance on strong ear protection and noise cancellation hardware by minimising acoustic noise at its source. Looping Star demonstrated sensitivity to the BOLD response in a complex, event‐related auditory fMRI paradigm, supporting its extension from simple blocked designs to complex cognitive tasks that are more widely used across studies. Optimisation and further characterisation with a range of paradigms and acquisition parameters is required to identify whether it indeed reveals additional information on cognitive processes involved in auditory processing. Furthermore, our study evaluated, for the first time, the test–retest reliability of Looping Star, which warrants further study to understand the impact of reduced scanner acoustic noise on cognitive strategies between sessions. Ultimately, Looping Star is a promising technique that offers a useful alternative to study the mechanisms of brain activity in sound averse populations.

## CONFLICT OF INTEREST

This work was conducted in collaboration with authors employed by GE Healthcare (Florian Wiesinger and Ana Beatriz Solana).

## Supporting information


**Appendix** **S1.** Supporting Information

## Data Availability

The scripts and toolboxes used in this research are available to download via the accompanying references. Please contact the corresponding author for access to specific scripts and data access, if collaboration is of interest.
