# Supplementary material for: Revealing the mechanisms behind novel auditory stimuli discrimination: An evaluation of silent functional MRI using looping star
Source: Hum Brain Mapp. 2021 Mar 17;42(9):2833–50. doi: 10.1002/hbm.25407 (PMC8127154; doi:10.1002/hbm.25407)
Supplement: Supplementary file 1 — Appendix S1. Supporting Information [file HBM-42-2833-s001.pdf]

Supplementary Material for Article:

Revealing the mechanisms behind novel auditory stimuli discrimination:

An evaluation of silent functional MRI using Looping Star

Corresponding Author:

Nikou Louise Damestani[1] - nikou.damestani@kcl.ac.uk

Co-authors:

Owen O'Daly[1], Ana Beatriz Solana[2] , Florian Wiesinger[1,2], David John Lythgoe[1], Simon Hill[1], Alfonso de Lara Rubio[1], Elena Makovac[1], Steven Charles Rees Williams[1], and Fernando Zelaya[1]

Institutions:

[1]Department of Neuroimaging, King's College London, London, United Kingdom

[2]ASL Europe, GE Healthcare, Munich, Germany

Full Institution Address:

[1]Centre for Neuroimaging Sciences, Institute of Psychiatry, Psychology and Neuroscience, King's College London, De Crespigny Park, Denmark Hill, London, United Kingdom, SE5 8AF

[2]GE Healthcare, Oskar-Schlemmer-Strasse 11, 80807, Munich, Germany

Key words:

silent functional MRI, Looping Star, novel sounds, auditory oddball, tone discrimination

A) Under-sampling the multi-echo fMRI Looping Star trajectory

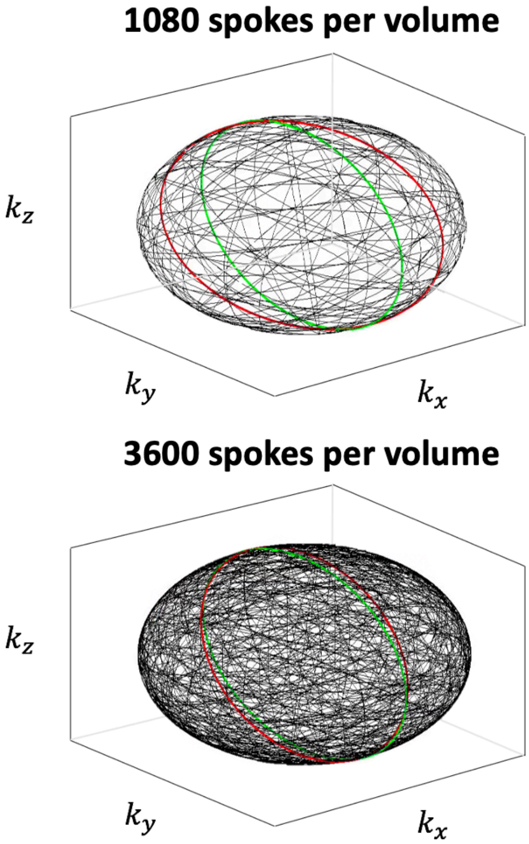

Figure A: Under-sampled 1080 spoke per volume (top) vs fully sampled 3600 spokes per volume (bottom) trajectories that can be used for a 3.2mm resolution and 19.2cm field-of-view in Looping Star. Full sampling refers to the number of spokes per volume being equivalent to the square of the matrix size. Red and green trajectory "loops" indicate pseudo-random orientations. k_x, k_y and k_z indicate the three dimensions of k-space. The under-sampled trajectory was used in this study for Looping Star fMRI to reduce repetition time.

B) Pre-processing Pipeline

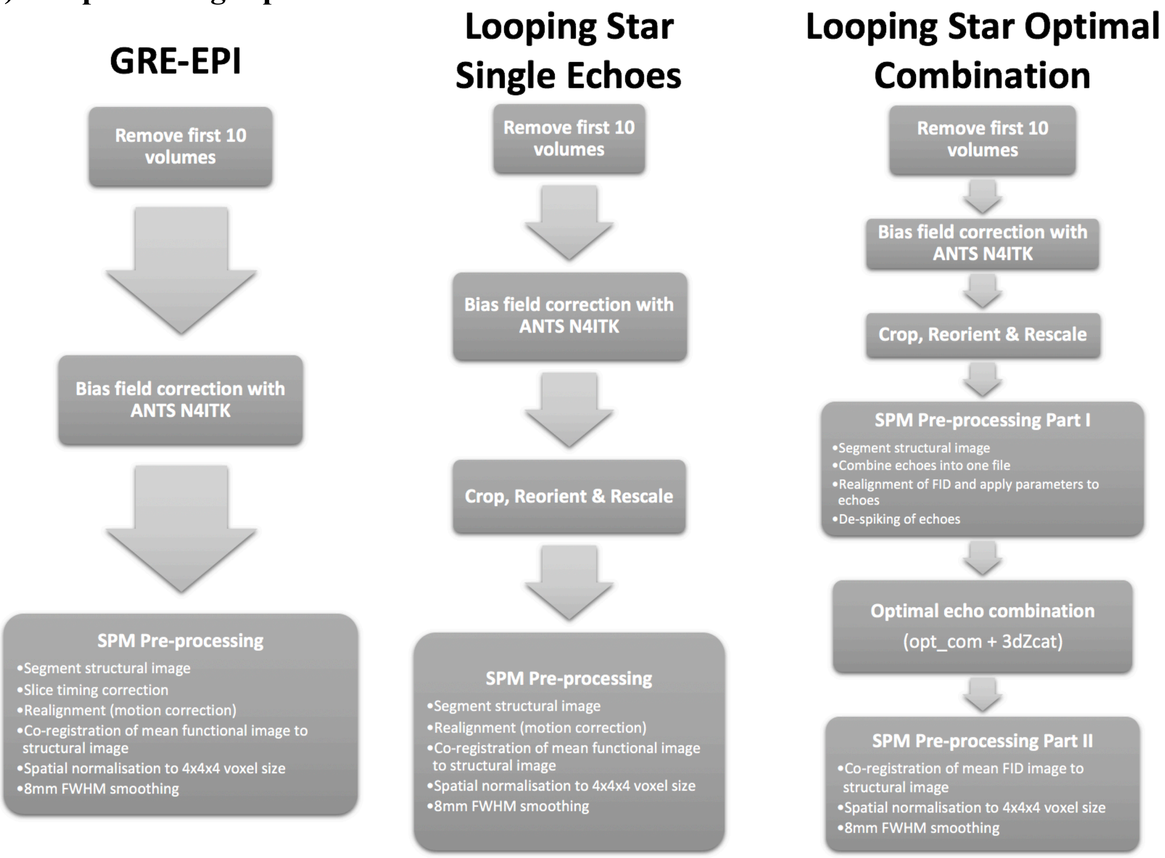

Figure B: Pre-processing pipeline workflow for each modality, also detailed with accompanying references for software used in Methods (Section 2.4). FID = free induction decay, FWHM = full-width, half-maximum.

C) Regions of Interest Visualisation

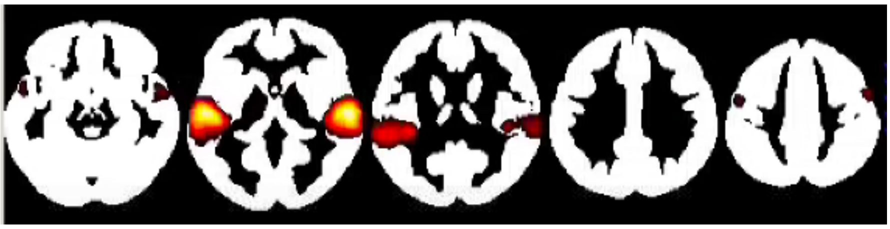

Figure C1: Grey matter mask (tissue prior template from SPM-12 software) with auditory region-of-interest (ROI) from Neurosynth for term "auditory" thresholded at z = 5 (Yarkoni et al, 2011) overlaid in red.

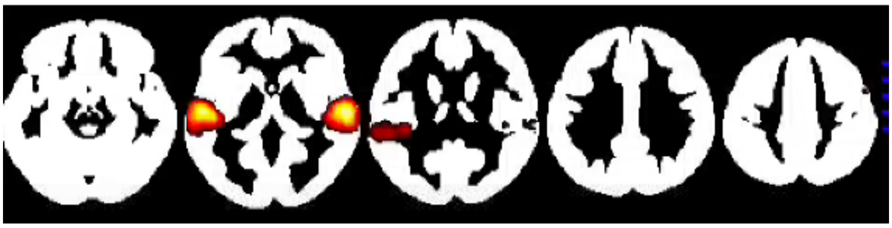

Figure C2: Grey matter mask (tissue prior template from SPM-12 software) with auditory region-of-interest (ROI) from Neurosynth for term "auditory" thresholded at z = 8 (Yarkoni et al, 2011) overlaid in red.

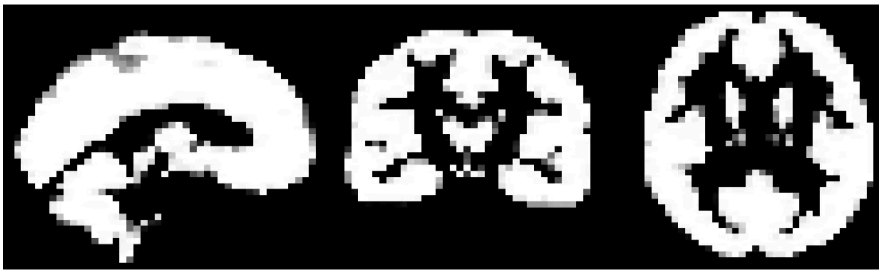

Figure C3: Grey matter mask (tissue prior template from SPM-12 software).

D) Raw Data Example

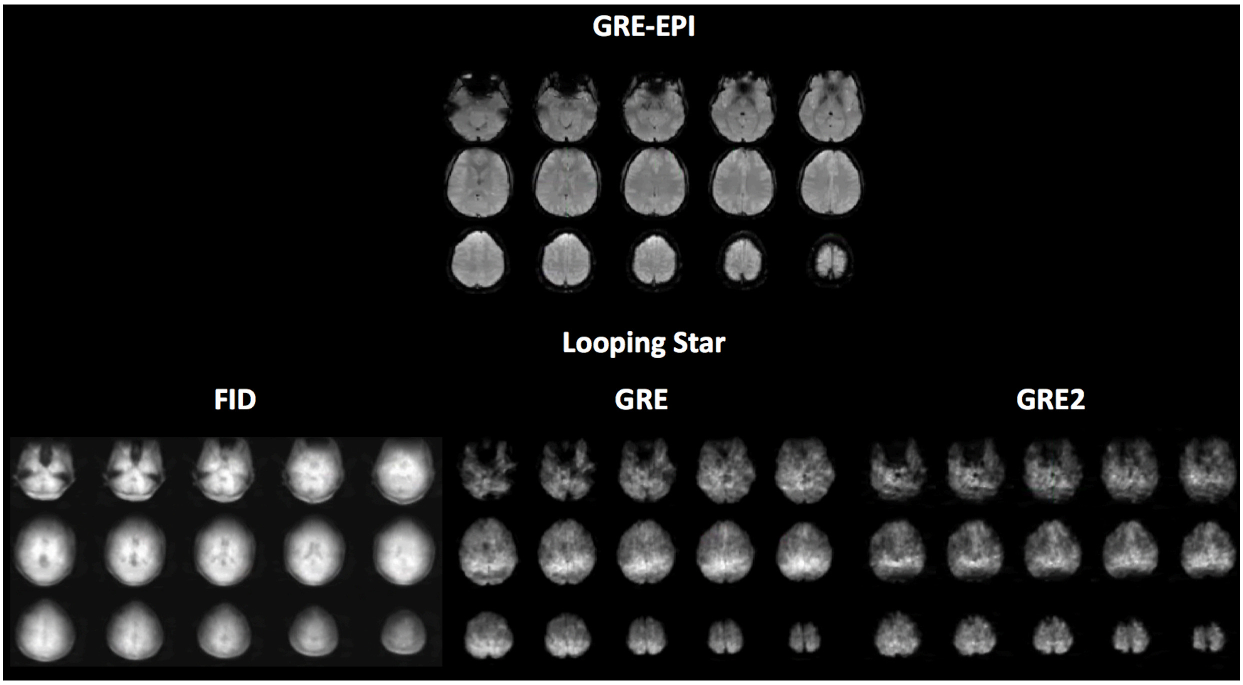

Figure D: Raw images of Looping Star individual echoes (free induction decay – FID, echo 1 – GRE, echo 2 – GRE2) and GRE-EPI data for one participant. Images shown prior to any reorientation, cropping, bias field correction or rescaling.

E) Behavioural & Physiological Results

Methods - Physiology

The physiological data from one participant in the second session was excluded due to severe artefacts in the pulse oximeter recording. First, an in-house MATLAB (Mathworks, 2019) script was used to locate the peaks in the pulse oximeter data and identify the inter-beat interval (IBI). The Kubios software (Tarvainen et al, 2014) was then used for threshold-based artefact correction, during which every IBI value is compared against a local average interval. If the IBI differed from the local average by more than 0.45 seconds ('very low' threshold in software), then it was identified as an artefact (Tarvainen et al, 2019). Kubios was also used to calculate the mean heart rate over a duration of 10 minutes, given these IBI values. The measure of interest was the mean heart rate over this duration, representative of the length of the paradigm, to identify any global changes in exertion.

Time per respiratory volume (or sampling period) was derived using the PhysIO toolbox within the TAPAS software in SPM-12 (Kasper et al, 2017). As stated in the software scripts and by Birn et al (2006), this is computed using filtered respiratory readings; interpolating the maximum and minimum breathing amplitudes between detected peaks and dividing them by interpolated durations between the maximum amplitudes of the breathing signals. The inputs included the TR, sampling interval of the physiological recording and number of scans per modality, with the slice to slice timing set as the TR for Looping Star given its three-dimensional acquisition. The mean and standard deviation for the sampling period were computed across the time series. Differences in the mean physiological measures from the different modalities and sessions were deemed statistically significant based on a simple two-sample T-test with equal variances assumed in Microsoft Excel with the Data Analysis Add-on, if the p-value (one-tailed) < 0.05 for a hypothesised difference $= 0$.

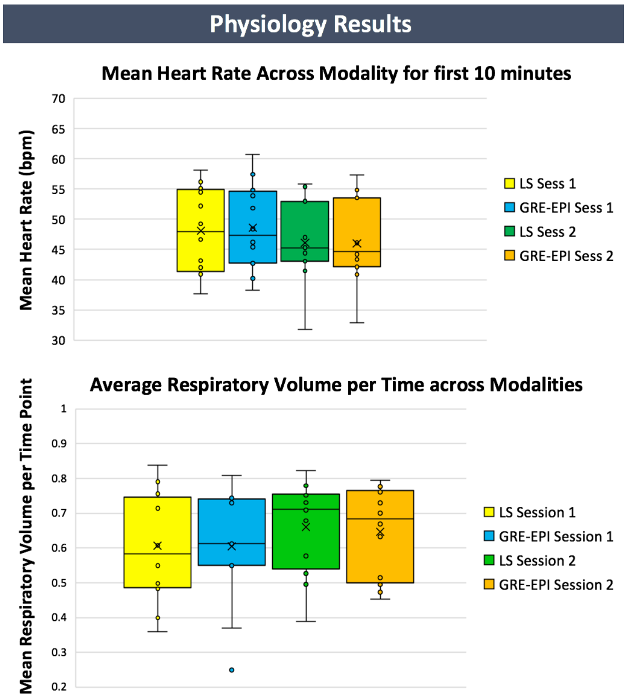

Figure E1: Physiology results for Looping Star (LS) and GRE-EPI for each session (Sess1 =. Session 1, Sess 2 = Session 2). (top) Mean heart rate across modality for duration 10 minutes. (bottom) Average respiratory volume per time point for each modality. No statistically significant differences identified.

Methods - Behaviour

Task performance accuracy was defined as whether a Novel or Deviant tone was correctly detected, according to the responses measured from the button box. Reaction time, between the onset of the tone and button press, was also recorded. The number of responses to Novel and Deviant tones that were accurate (< 625ms from onset) were summed with those that were late (> 625ms but < 1250ms). This was performed as the interval between tone onsets was very short, meaning fast reaction times were required: upon inspection of the behavioural data, we found many responses that appeared within the following onset (i.e. between 625ms and 1250ms after the previous onset), therefore indicating an accurate but delayed response. We also explored the accuracy before and after the Standard and Deviant tones were swapped. Simple paired t-tests were performed using the Microsoft Excel Data Analysis add-on, with alpha = 0.05 and a hypothesised difference of zero. Results were deemed statistically significant if the p-value (one-tailed) < 0.05.

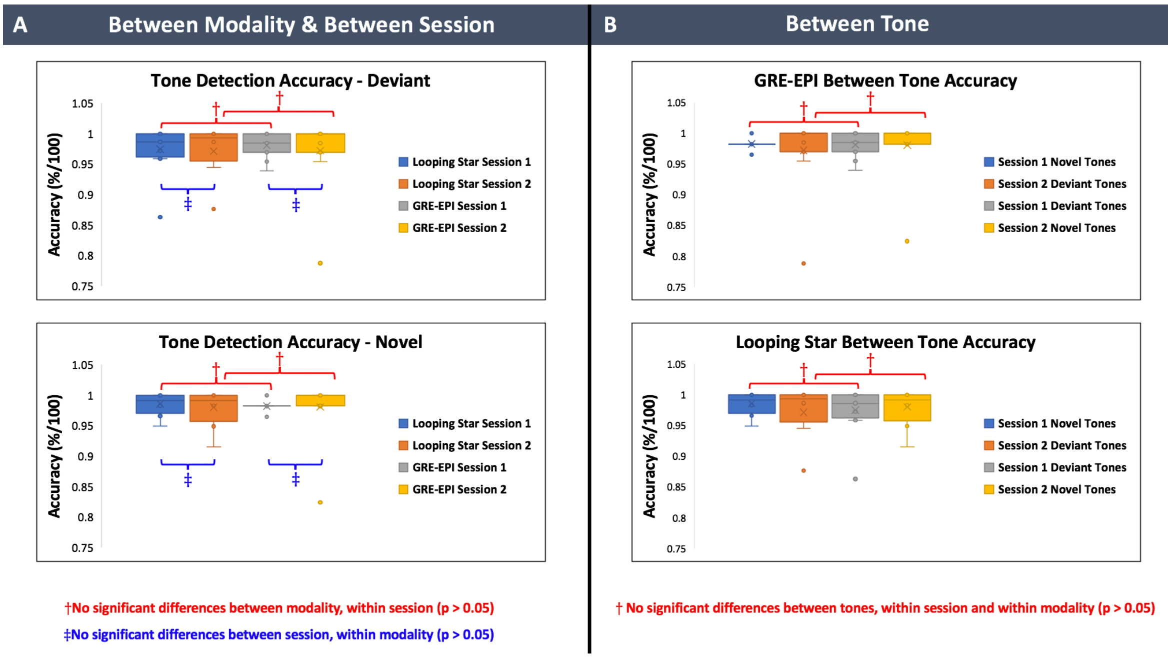

Figure E2: Behavioural results summarised for Looping Star and GRE-EPI. A) Between modality and between session tests are summarised. B) Between tone (Novel and Deviant) differences within session and within modality tests are summarised. No significant differences were found for any groups of tests (represented by parentheses).

Table E1: Behavioural results summary of accuracy values, purple font indicates highest percentage accuracy across modality and session for each tone.

Session Type	**Number of Deviant Tones Accurately Detected**	**Number of Novel Tones Accurately Detected**	**Number of Deviant Tones Detected >625ms Late**	**Number of Novel Tones Detected >625ms Late**	**Total Deviant Tones Detected**	**Total Novel Tones Detected**
GRE-EPI Session 1	57.8 ± 9.3 /66	51.5 ± 7.0 /57	7.1±8.8 /66	4.5±7.2 /57	64.8±1.4 /66 **(98.2%)**	56.0±0.6 /57 **(98.2%)**
Looping Star Session 1	67.3 ± 3.3 /73	55.7 ± 2.6 /59	**3.9±2.9 /73**	**2.5±2.2 /59**	71.2±2.8 /73 **(97.5%)**	58.2±1.0 /59 **(98.6%)**
GRE-EPI Session 2	58.0 ± 7.7 /66	51.9 ± 8.0 /57	6.2±6.1 /66	4.0±7.2 /57	64.1±3.9 /66 **(97.2%)**	55.9±2.8 /57 **(98.1%)**
Looping Star Session 2	65.2 ± 7.8 /73	53.0 ± 6.4 /59	5.8±7.0 /73	4.8±6.0 /59	70.9±3.4 /73 **(97.1%)**	57.8±1.6 /59 **(98.0%)**
Pre-scan Test (Session 1)	34.6 ± 6.4 /41	22.6 ± 3.0 /25	3.8±3.4 /41	1.9±2.3 /25	38.3±3.7 /41 **(93.5%)**	24.5±1.2 /25 **(98.0%)**

Table E2: Overall sum of the number of missed tones across participants for each modality and session, before and after the Standard and Deviant tones were swapped (half-way through the paradigm). There was no significant difference (i.e. p(one-tailed) > 0.05) between the overall number of tones missed before and after the swap for all modalities, using a simple paired T-test.

Session Type	**Number of Novel Tones Missed Before Swap**	**Number of Novel Tones Missed After Swap**	**Number of Deviant Tones Missed Before Swap**	**Number of Deviant Tones Missed After Swap**
GRE-EPI Session 1	5	8	1	8
Looping Star Session 1	4	6	12	10
GRE-EPI Session 2	4	7	8	11
Looping Star Session 2	8	2	14	8
**Total number of missed tones across modality**	**21**	**23**	**35**	**37**

References

Birn, R.M., Diamond, J.B., Smith, M.A. and Bandettini, P.A. (2006). Separating respiratory-variation-related fluctuations from neuronal-activity-related fluctuations in fMRI. *Neuroimage*, 31(4), pp.1536-1548. https://doi.org/10.1016/j.neuroimage.2006.02.048

Kasper, L., Bollmann, S., Diaconescu, A.O., Hutton, C., Heinzle, J., Iglesias, S., Hauser, T.U., Sebold, M., Manjaly, Z.M., Pruessmann, K.P., & Stephan, K.E. (2017). The PhysIO toolbox for modeling physiological noise in fMRI data. *Journal of neuroscience methods*, 276, pp.56-72. https://doi.org/10.1016/j.jneumeth.2016.10.019

Mathworks. MATLAB. Accessed October 2019. Version used varied depending on compatibility with software and toolboxes.

Tarvainen, M.P., Niskanen, J.P., Lipponen, J.A., Ranta-Aho, P.O. and Karjalainen, P.A. (2014). Kubios HRV–heart rate variability analysis software. *Computer methods and programs in biomedicine*, 113(1), pp.210-220. https://doi.org/10.1016/j.cmpb.2013.07.024

Tarvainen, M.P., Niskanen, J.P., Lipponen, J.A., & Ranta-Aho, P.O. (2019). Kubios HRV (ver. 3.3). User's Guide. Accessed at https://www.kubios.com/support/ .

Yarkoni, T., Poldrack, R.A., Nichols, T.E., Van Essen, D.C. and Wager, T.D. (2011). Large-scale automated synthesis of human functional neuroimaging data. *Nature methods*, 8(8), p.665. https://doi.org/10.1038/nmeth.1635 . Accessed at https://neurosynth.org
